# Gene Regulation Analysis Reveals Perturbations of Autism Spectrum Disorder during Neural System Development

**DOI:** 10.3390/genes12121901

**Published:** 2021-11-27

**Authors:** Dan Li, Joshua Xu, Mary Qu Yang

**Affiliations:** 1 Division of Bioinformatics and Biostatistics, National Center for Toxicological Research, U.S. Food and Drug Administration, Jefferson, AR 72079, USA; dan.li@fda.hhs.gov; 2 MidSouth Bioinformatics Center, Joint Bioinformatics Graduate Program of University of Arkansas at Little Rock, University of Arkansas for Medical Sciences, Little Rock, AR 72204, USA

**Keywords:** autism spectrum disorder (ASD), neural progenitor cells (NPCs), pathway, regulatory cascades, regulatory variations

## Abstract

Autism spectrum disorder (ASD) is a neurodevelopmental disorder that impedes patients’ cognition, social, speech and communication skills. ASD is highly heterogeneous with a variety of etiologies and clinical manifestations. The prevalence rate of ASD increased steadily in recent years. Presently, molecular mechanisms underlying ASD occurrence and development remain to be elucidated. Here, we integrated multi-layer genomics data to investigate the transcriptome and pathway dysregulations in ASD development. The RNA sequencing (RNA-seq) expression profiles of induced pluripotent stem cells (iPSCs), neural progenitor cells (NPCs) and neuron cells from ASD and normal samples were compared in our study. We found that substantially more genes were differentially expressed in the NPCs than the iPSCs. Consistently, gene set variation analysis revealed that the activity of the known ASD pathways in NPCs and neural cells were significantly different from the iPSCs, suggesting that ASD occurred at the early stage of neural system development. We further constructed comprehensive brain- and neural-specific regulatory networks by incorporating transcription factor (TF) and gene interactions with long 5 non-coding RNA(lncRNA) and protein interactions. We then overlaid the transcriptomes of different cell types on the regulatory networks to infer the regulatory cascades. The variations of the regulatory cascades between ASD and normal samples uncovered a set of novel disease-associated genes and gene interactions, particularly highlighting the functional roles of *ELF3* and the interaction between *STAT1* and lncRNA *ELF3-AS* 1 in the disease development. These new findings extend our understanding of ASD and offer putative new therapeutic targets for further studies.

## 1. Introduction

Autism spectrum disorder (ASD) is a neurodevelopmental disorder that impedes patients’ cognition, social skills, speech and communication [1,2]. Autism can be diagnosed at any age; however, symptoms usually appear in the first two years of life [3]. ASD is a complex and heterogeneous condition affecting an increasing number of children in the U.S. A new estimate announced by the Centers for Disease Control and Prevention (CDC) indicates that ASD affected one in 59 (1.7%) individuals in 2018, an increase from one in 68 (1.5%) just two years earlier [4]. Primary autism, called idiopathic ASD of unknown causes, accounts for about 85% of cases [5]. In contrast, secondary ASD accounting for 15% of patients, has specific causes such as down syndrome, Fragile X syndrome and tuberous sclerosis [6,7,8]. The high complexity, heterogeneity and wide variability, described by the term spectrum, make this condition challenging to study and treat.

Currently, considerable research efforts at the molecular level have identified ASD-associated genes [1,2,9,10,11,12]. Voineagu et al. identified several gene modules and the convergent molecular abnormalities in ASD by investigating the differentially expressed genes (DEGs) and co-expression patterns [2]. Skafidas et al. applied genome-wide association studies (GWAS) to discover the candidate genes related to ASD and identified several significant pathways [9]. A number of long non-coding RNAs (lncRNAs) that were anomalously expressed in ASD were identified, indicating their potential roles in the disorder [12]. Furthermore, the dysregulation of gene expression was reported to be associated with cell production, DNA-damage response, and cell cycle functions in ASD cases [13,14].

Marchetto et al. [1] modeled ASD utilizing induced pluripotent stem cell (iPSC) technology for studying disease mechanisms. They reprogrammed fibroblasts from idiopathic ASD patients with macrencephaly and control subjects with typically developing individuals to generate induced iPSCs, neural progenitor cells (NPCs) and neurons. They found that increased proliferation rates in ASD-derived NPCs may be responsible for early overgrowth in ASD patients, while abnormal neurogenesis and reduced synaptogenesis in ASD-derived neurons may lead to functional defects in neuronal networks. Their study also found that drug IGF-1 showed a therapeutic potential for activating neuronal spikes and rescuing defects in the neuronal networks.

In this study, we developed a regulatory network analysis approach to investigate the dynamic changes of transcriptome during ASD development. The RNA-seq data of three cell types at the different cell differentiation stages, namely iPSCs, NPCs and neuron cells reported in the previous study [1], were systematically analyzed and compared. Novel disease genes including transcription factors (TFs), target protein-coding genes (PCGs) and lncRNAs such as *IRF1*, *ELF3* and *ELF3-AS1*, and their roles in regulations of ASD were discovered by our study. These new findings advance our understanding of the disorder and provide new insights for further studies.

## 2. Results

### 2.1. Differential Expression and Pathway Analyses Highlighted the NPC Stage of ASD

We downloaded RNA-seq data (GSE67528) generated based on three cell types, including iPSCs (11 normal, 17 ASD), NPCs (nine normal, 21 ASD) and neuron cells (eight normal, 17 ASD); each cell type contains ASD and normal samples [1]. The iPSCs were transduced from the skin fibroblasts of ASD and normal subjects, then were derived into NPCs and neuron cells [1].

**Figure 1:**
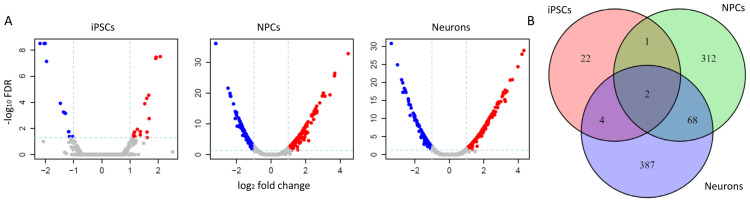
Differential expression analysis in three cell types. (**A**) volcano plots showed the significant DEGs with cutoff |log2FC|> 1 and FDR < 0.05. Blue points represent unexpressed genes, while red points refer to overexpressed genes in different neural cells types. (**B**) The unique and common DEGs among the three cell types demonstrated the distinct gene expression change patterns in iPSCs compared to NPCs and neural cells.

Differential expression (DE) analysis was performed on each cell type between ASD and normal samples. As a result, 29 significant DEGs (25 PCGs, four lncRNAs) were found in iPSCs, whereas 383 (341 PCGs, 42 lncRNAs) and 461 DEGs (430 PCGs, 31 lncRNAs) were found in NPCs and neuron cells (Figure 1A, Methods), respectively. A substantially increasing number of DEGs in NPCs compared to iPSCs (383 versus 29) indicated that the initiation and development of ASD occurred during cell differentiation from iPSC to NPC. Moreover, only 70 DEGs (67 PCGs, three lncRNAs) were shared by NPC and neuron cells (Figure 1B), representing 18.2% (70/341) and 15.2% (70/461) of DEGs of these two cell types. Additionally, none of the known signal pathways were enriched of these common DEGs, suggesting distinct mechanisms underlying the neurodevelopment of ASD at the NPC- and neuron- stages. The differentially expressed lncRNAs were listed in Supplementary Table S1.

The enriched functional annotations, based on the DEGs of each cell type, were then identified via the Database for Annotation, Visualization and Integrated Discovery (DAVID) [15]. The 29 iPSC DEGs were related to four GO biological process terms, including the cerebral cortex and subpallium development. Two genes, DLX1 and DLX2, were involved in these two functions and were significantly downregulated in ASD samples (log2FC = −2.05 and log2FC = −1.96, respectively). Moreover, several ASD-related KEGG pathways, including calcium signaling and focal adhesion, reported by previous studies [9,16] were identified as using DEGs of the NPC and neuron cell types (Table 1).

**Figure 2:**
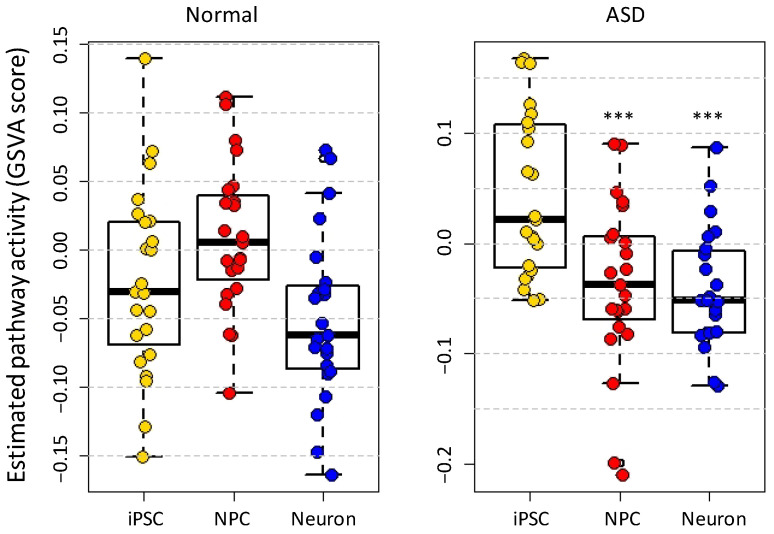
The estimated activity of 23 ASD-associated pathways in normal and ASD samples in three neural cell types, representing different stages of cell development. The pathway activity was assessed based on GSVA scores. In the ASD samples, the differences of GSVA scores between iPSC and NPC (*p*-value = 7.64 × 10^−4^), as well as between iPSC and neuron cells (*p*-value = 7.11 × 10^−5^) were statistically significant. *** p<0.001, two-tail Student’s *t*-test.

Next, we calculated the gene set variation analysis (GSVA) scores [17] for the 23 ASD-associated KEGG pathways [9] (Supplementary Figure S1) representing their relative activation status in the three cell types (Figure 2). The activation status did not change significantly from iPSC to NPC (*p*-value = 0.0789) and neuron cell (*p*-value = 0.166) stages in normal samples. In contrast, the differences in GSVA scores between iPSC and NPC (*p*-value = 7.64 × 10^−4^), and iPSC and neuron cells (*p*-value = 7.11 × 10^−5^) were statistically significant in the ASD samples. Many pathways have negative GSVA scores starting from the NPC stage, especially in ASD samples indicating that the expressions of the involved genes were changed compared to iPSC (Methods).

**Figure 3:**
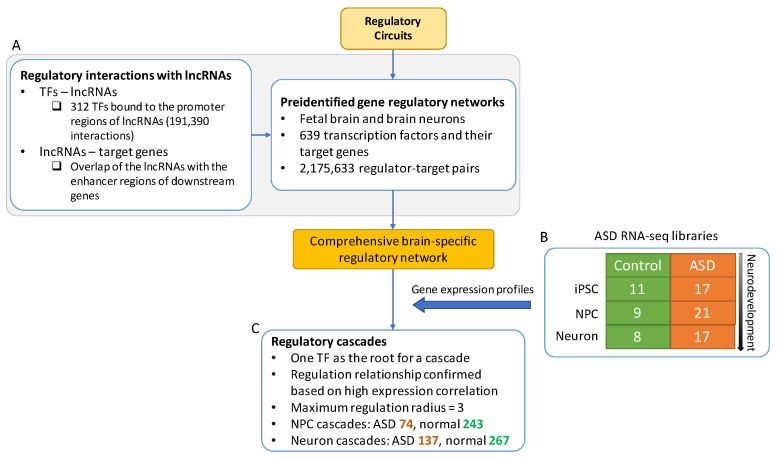
The workflow of generating gene regulatory cascades. The gene regulatory networks of fetal brain and brain neuron tissues were created based on Regulatory Circuits [18] and lncRNAs were incorporated to complete the regulatory networks (**A**). The gene expression profiles (**B**) of ASD were overlaid on the network to establish ASD regulatory cascades (**C**). The root of each regulatory cascade is a TF.

Then we conducted a regulatory network analysis that focuses on the regulation variations caused by ASD in different stages of neurodevelopment. The main components of the analysis were illustrated in Figure 3 and described in the following sections.

### 2.2. Construct Brain- and Neural-Specific Regulator-Target Regulation Pairs

We further studied the regulation variations caused by ASD in the three neural cell types. First, we obtained the gene regulatory networks of the fetal brain and brain neurons from Regulatory Circuits [18]. Regulatory Circuits is a large database containing comprehensive cell type- and tissue-specific regulatory networks constructed computationally. Based on 639 TFs and their target PCGs, we extracted 2,175,633 regulator-target regulation pairs from the networks. After excluding low expressed genes, the remaining regulation pairs consisting of 424 TFs and 11,435 brain- and neuron-specific target genes were obtained.

In addition, we incorporated lncRNAs into the regulatory networks. The position weight matrices (PWMs) from the JASPAR, a database of TF binding profiles, were used to search the TF-lncRNA regulations based on motif matching [19]. Consequently, we found 312 TFs that bound to the promoter regions of the lncRNAs using MOtif Occurrence Detection Suite (MOODS), a fast motif matching approach [20]. A total of 191,390 significant matched bindings (*p*-value < 5 × 10^−5^) between the TFs and lncRNAs were identified. We then inferred the downstream genes that were potentially regulated by the lncRNAs via identifying overlap of the lncRNAs with the enhancer regions of the potential target genes. These predicted interactions were used to complete the list of the brain- and neural-specific regulator-target pairs for the subsequent analysis. (Figure 3A, see Methods for details).

### 2.3. Expression Correlations between Regulators and Target Genes

We assessed the expression correlation of the regulator-target pairs in individual cell types based on Pearson correlation coefficients (Figure 3B). More coordinately expressed regulator-target pairs were found in normal samples than ASD samples of the NPCs and neuron cells under the correlation coefficient cutoff at 0.75. Interestingly, we identified fewer correlated regulator-target pairs in normal samples than in ASD samples of iPSC cells. The regulator-target regulatory relationships were derived from fetal brain and brain neurons; epigenetic differences of the stem cells may contribute to this observation. Comparable results were observed in the lncRNA-involved regulations (Supplementary Table S2).

We hypothesized that transcription dysregulation reflects mechanisms underlying ASD development and can provide further insights into the signaling pathways involved in the disease. Thus, we investigated regulatory interactions newly gained in the ASD samples in comparison to normal samples. Based on the regulator-target coordinately expressed patterns (correlation cutoff = 0.75), we identified 462 and 974 unique target genes presented in ASD samples only of NPC and neuron cell types, respectively. Functional annotations of these two gene groups revealed several significant pathways (Table 2), showing their involvement in ASD.

Similar to the results of DE analysis, the target genes that we identified in ASD samples of NPCs were enriched of many ASD-associated pathways [9,11,21]. For example, a newly published study about the relationship between melatonin and ASD during fetal development suggested that neuroprotective and circadian entraining (Table 2) would help reduce the risk of neurodevelopmental disorders [21]. Collectively, our results showed that transcriptional dysregulation and pathway perturbation occurred as early as the NPC stage during the ASD development.

### 2.4. Construction of Regulatory Cascades of ASD

We constructed tree-like regulatory cascades consisting of PGCs and lncRNAs to further investigate regulation changes in ASD (Figure 3C). Starting from the individual transcription factors, target genes were added based on the brain- and neural-specific regulator-target regulations and restrictive criteria including gene expression correlation, motif matching, and enhancer overlap. A maximum regulation radius was set at three to maintain the cascade size (see Methods for details). Substantially more regulatory cascades were found in normal samples than ASD, which were 243 versus 74 and 267 versus 137 cascades in NPCs and neural cells, respectively. The sizes of cascades were also much larger in normal samples compared to ASD (Supplementary Figure S2), suggesting that many regulations were lost due to ASD starting at the early stage of the disease.

Among 243 regulatory cascades in normal NPC, 26 of them containing over 1000 genes were lost in ASD (Supplementary Figure S3). The root TFs of those cascades were enriched in regulation of growth (*p*-value = 3.7 × 10^−5^), positive regulation of cell population proliferation (*p*-value = 3.2 × 10^−4^), regulation of transforming growth factor beta 3 production (*p*-value = 3.1 × 10^−5^). Hence, these lost TF regulatory cascades may be responsible for the increased brain size of ASD patients in the early stage of the disease. These TFs were considered ASD-associated and the enriched pathways of these 26 cascades were listed in Supplementary Table S3.

Of 267 cascades in normal neural cells, eight and 89 of them containing over 1000 and 20 genes, respectively, were lost in ASD neural cells (Supplementary Figure S4). The root TFs of the eight cascades were abundant in positive regulation of neural cell death (*p*-value =1.05 × 10^−5^), while the TF of the 89 cascades were enriched in peripheral nervous system development (*p*-value = 4.9 × 10^−4^), nerve development (*p*-value = 1.02 × 10^−4^), and neuron differentiation (*p*-value = 1.7 × 10^−7^).

**Figure 4:**
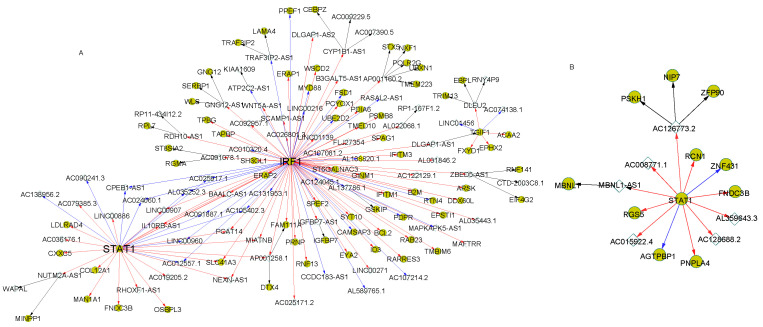
The regulatory cascades with transcription factor *STAT1* as the root in the neuron cells. The expression correlation coefficients of connected genes were larger than 0.8 in the cascades. (**A**) showed the regulatory cascade in the normal samples. (**B**) represented the cascade with the root gene *STAT1* in the ASD samples. The gold circles denoted PCGs and the white diamonds represent lncRNAs. The directed arrows indicate the regulations from the regulators to the target genes with diverse correlation patterns: Positive (red), negative (blue) and lncRNA-target based on enhancer matching (black).

Signal transducer and activator of transcription 1 (*STAT1*) is one of the ASD-associated genes. It was reported that *STAT1* could respond to cytokine and some growth factors and play critical roles in ASD [22]. In the regulatory cascade rooted at *STAT1*, more regulations were presented in the normal cascade than in ASD for the neural cells (Figure 4). *IRF1*, another ASD-associated gene that communicates with *STAT1* in ASD [23], was found to regulate many target genes in this regulatory cascade (Figure 4A). The target PGCs in the regulatory cascade of normal samples were enriched of two ASD-associated pathways: Toxoplasmosis (*p*-value = 0.012), protein processing in the endoplasmic reticulum (*p*-value = 0.03) and the GO term: Endoplasmic reticulum (*p*-value = 2.2 × 10^−6^). The relationships between toxoplasmosis, in which *STAT1* and *IRF1* were highly involved, and ASD have been widely studied [10,23,24]. Our results suggested the regulation interaction loss between *STAT1* and *IRF1*, and the relevant target genes in ASD cells may attribute to the ASD development.

**Figure 5:**
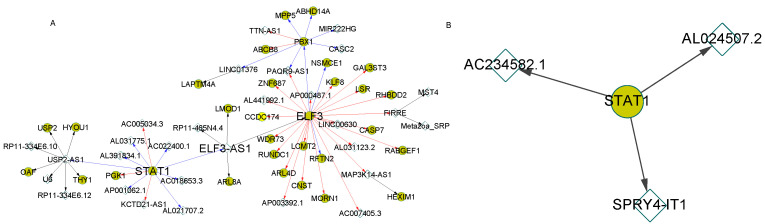
The regulatory cascades with transcription factor *STAT1* as the root in NPCs. The expression correlation coefficients of connected genes were larger than 0.8 in the cascades. (**A**) showed the regulatory cascade in the normal samples. (**B**) represented the cascade with the root gene *STAT1* in the ASD samples.

In the NPC stage, different regulatory cascades with similar patterns were observed with *STAT1* as the root. A larger cascade was found in normal samples compared with ASD (Figure 5). In particular, lncRNA *ELF3-AS1*, an antisense RNA of *ELF3*, was identified by motif matching to be regulated by *STAT1* (Supplementary Figure S5). This regulation was further supported by the negative expression correlation (r = −0.809) between *STAT1* and *ELF3-AS1* in NPC.

To the best of our knowledge, there is no article reporting the involvement of *ELF3* in ASD. A previous study of mouse NPC claimed that *ELF3* could be a novel isoform of β-G spectrin, which has potential roles in neural stem cell development. *ELF3* was also considered to be an axonal sprouting marker during embryonic development [25]. In this study, we found that the regulation between *STAT1* and *ELF3* as well as the other target genes of *STAT1* were lost in ASD neural progenitor cells (NPCs), indicating disorder of the axonal sprouting functions in the ASD and its association with ASD.

## 3. Materials and Methods

### 3.1. RNA-Seq Data Process and Differential Expression Analysis

We downloaded 83 RNA-seq libraries (GSE67528) including 28 controls and 55 ASD samples from [1]. In the original study [1], the skin fibroblasts from eight idiopathic ASD individuals displayed brain overgrowth early in life and five age/gender-matched control individuals were reprogrammed to produce three cell types, iPSCs, NPCs and neurons, along with the neuron development. The RNA-seq data were then were processed to calculate the gene expression profiles. The unexpressed genes (median read counts less than 20) were first removed from our study. A total of 16,950 genes, including 14,667 PCGs, 953 lincRNAs, and 1330 antisense RNAs, remained for further analysis. Annotations of lncRNA and antisense RNA were collected from Ensembl (GRCh38.90) [26]. Gene expression profiles were calculated using Cufflinks [27] and HTSeq-count [28]. The R package, edgeR [29], was applied for differentially expressed genes identification between normal and ASD samples. The criteria |log2FC|>1 and FDR<0.05 were employed for significance measurement.

### 3.2. Pathway Activation Assessment via GSVA Score

The member genes of the 23 ASD-associated pathways that were highly expressed in our data were used to calculate GSVA scores using the R package GSVA [17]. We calculated the difference between the largest positive and negative deviations on the normalized gene expression log2(geneCount+1), and applied default arguments. Finally, the median GSVA score of each pathway (gene set) was obtained based on the cell types representing the activation status.

### 3.3. lncRNA-Involved Regulations

A total of 537 position weight matrices (PWMs) for motifs of the transcription factors were downloaded from JASPAR 2018 [19]. DNA sequences of the promoter regions (−1000 bp to +200 bp respective to transcription start sites) of the lncRNAs were extracted from the human genome (version hg38). Next, these motifs were aligned with the promoter regions via MOODs. The statistical measure was calculated for the identification of the significant motif matches. A much stricter threshold (*p*-value = 5 × 10^−5^) was applied since this *p*-value was calculated once for multiple short sequences of the promoter regions. The motif matches with the *p*-value under the threshold were output and indicated the TF-lncRNA regulations.

To discover the potential target genes regulated by lncRNAs, we matched the lncRNAs with the enhancer regions of the target genes. The enhancer regions, based on the fetal placenta and fetal spinal cord tissues, were downloaded from the EnhancerAtlas. The original enhancer regions under human genome version hg19 were converted to hg38 using the UCSC liftover tool [30]. A potential target gene of the given lncRNA was discovered when there was an overlap of the genomic coordinates of the target gene’s enhancer and the lncRNA. These lncRNA-target regulations were not further confirmed by the gene expression correlation because they physically overlapped and the lncRNAs were highly expressed.

### 3.4. Regulatory Cascade Construction

Beginning with an ASD-associated transcription factor as the root of the regulatory cascade, we first calculated the gene expression correlation between this TF and its target genes listed in our reference regulation list. The target genes were linked to the TF as layer one children in the cascade if the correlation coefficients were higher than 0.8. Next, the cascade grew by adding new target genes regulated by the layer one genes. Here, up to three layers were allowed. PARTHER [31] was used for biological process enrichment analysis for root TFs of the regulatory cascades.

## 4. Discussion

Using genomic analyses focusing on ASD and neural system development, we identified many ASD-associated genes and pathways enriched in the neural progenitor cells, which represent an intermediate stage in development between iPSC and neuron cells. Our observations indicated that ASD might occur during the development from iPSC to NPC stages. The genes that we identified based on this cell type could be potential targets guiding further ASD studies.

Among the ASD-associated genes accumulated through many prior studies, only a few were differentially expressed in our data. For instance, 873 ASD-associated genes reported by AutDB were highly expressed in our data, and only three significant DEGs were identified between iPSC ASD and iPSC normal samples. The number of DEGs among AutDB genes in NPCs and neuron cells were only 32 and 19, respectively. Similarly, 265 ASD-associated rare SNVs were identified using the same RNA-seq date [1]. Of these, only six (*EGR2*, *KCNJ5*, *MYO18B*, *NTN1*, *SERPINE1* and *ZSCAN10*) and five (*CCDC33*, *EGFLAM*, *ITLN2*, *MAPK15* and *NTN1*) were DEGs found in NPC and neuron cell samples, respectively, while no DEGs were found in iPSC. In this case, we extended the analyses onto the regulation variations to study the changes in the regulatory relationships between genes perturbed by ASD. By constructing the regulatory cascades of ASD, we discovered a set of novel disease associated genes including lncRNAs.

Our work revealed the potential involvement of *ELF3*, *ELF3-AS1* in ASD development which has not been widely reported. *ELF3* was found to be associated with mouse neural stem cell development [25]. A recent study identified *ELF3* as one of the top ranked active TFs that are important for neural induction based on expression profiles at the different time points of the induction process as well as ChIP-Seq experiments [32]. Our work further demonstrated the importance of this gene in neural development. Based on regulatory cascade analysis, we found that *ELF3* was interacted with *ELF3-AS1* and *STAT1* in normal NPCs, while those interactions were lost in ASD samples. Hence, *ELF3* and its interactors may have essential roles in the ASD progression.

Over the past decade, though hundred of genes related to ASD have been reported, these genes only account for 10–20% of autism cases [33]. Our regulatory network analysis centered at TFs provides a unique way to reveal master regulators, which position at the top of regulatory hierarchies and control the transcriptional activities of many downstream genes. For instance, we found some TFs regulate over 1000 genes in the regulatory cascades. It has been shown that a TF or a set of TFs can drive the development of diseases and biological processes [34,35,36,37]. Hence, the ASD-associated TFs identified by our work can offer new therapeutic targets, leading to more effective treatments for more autism patients. Our regulatory analysis started with the extraction of transcriptional regulatory circuits from Regulatory Circuits database. The database was built through genome-wide mapping promotes and enhancers, and their linkages with TFs and target genes to data of FANTOM5 consortium [18,38,39]. Precisely and comprehensively locating regulatory regions, particularly for enhancers, is a challenging task. Enhanced knowledge of these regulatory regions will help improve our model to achieve better results. The ASD regulatory cascades that we established in this study offer a framework for revealing new disease-related genes and can be applied and extended to study other tissues and diseases.

## 5. Conclusions

We investigated alterations of gene expression, pathways and regulatory interactions to understand ASD initialization and progression. Through the systematical study of the dynamic changes of transcriptomes and regulatory networks in three neural cell types, we found that abnormal gene expression and activity of ASD-related signal pathways consistently occurred at NPCs, suggesting that transcriptional dysregulation of ASD at the early stage of the neural system development. Our regulatory cascade analysis revealed that many regulations between TFs and downstream targets were lost in ASD compared to normal samples. Hence, the TFs of these lost cascades may have crucial roles in the neural system and brain development. In particular, we discovered *STAT1* and lncRNA *ELF3-AS1* as novel genomic elements related to ASD development. Our findings enhance our understanding of ASD at the regulation level.

## Figures and Tables

**Table 1:** Functional annotations of the DEGs of different cell types in neural system development.

iPSC	Term	*p*-Value	Benjamini
GOTERM_BP_DIRECT	cerebral cortex GABAergic interneuron fate commitment	2.5 × 10^−3^	0.48
GOTERM_CC_DIRECT	endoplasmic reticulum	2.6 × 10^−3^	0.16
GOTERM_BP_DIRECT	subpallium development	3.7 × 10^−3^	0.39
GOTERM_BP_DIRECT	regulation of transcription from RNA polymerase II promoter involved in forebrain neuron fate commitment	3.7 × 10^−3^	0.39
**NPC**	**Term**	***p*** **-Value**	**Benjamini**
KEGG_PATHWAY	Neuroactive ligand-receptor interaction *	1.9 × 10^−3^	0.3
KEGG_PATHWAY	Pathways in cancer	2.5 × 10^−3^	0.21
KEGG_PATHWAY	Focal adhesion *	51 × 10^−3^	0.27
KEGG_PATHWAY	Calcium signaling pathway *	6.1 × 10^−3^	0.25
KEGG_PATHWAY	Retrograde endocannabinoid signaling	1.1 × 10^−2^	0.34
KEGG_PATHWAY	Wnt signaling pathway *	1.4 × 10^−2^	0.36
KEGG_PATHWAY	Regulation of lipolysis in adipocytes	0.02	0.42
KEGG_PATHWAY	PI3K-Akt signaling pathway *	2.7 × 10^−2^	0.47
**Neuron**	**Term**	***p*** **-Value**	**Benjamini**
KEGG_PATHWAY	ECM-receptor interaction	1.7 × 10^−13^	2.9 × 10^−11^
KEGG_PATHWAY	Protein digestion and absorption	2.8 × 10^−12^	2.3 × 10^−10^
KEGG_PATHWAY	Focal adhesion *	2 × 10^−9^	1.1 × 10^−7^
KEGG_PATHWAY	PI3K-Akt signaling pathway *	3 × 10^−9^	1.3 × 10^−7^
KEGG_PATHWAY	Amoebiasis	4.2 × 10^−4^	1.4 × 10^−2^
KEGG_PATHWAY	Neuroactive ligand-receptor interaction *	8.2 × 10^−4^	2.3 × 10^−2^
KEGG_PATHWAY	TGF-beta signaling pathway	2 × 10^−3^	4.8 × 10^−2^
KEGG_PATHWAY	Renin-angiotensin system	1.2 × 10^−2^	0.23
KEGG_PATHWAY	Platelet activation	2.1 × 10^−2^	0.33
KEGG_PATHWAY	Hypertrophic cardiomyopathy (HCM)	2.5 × 10^−2^	0.35
KEGG_PATHWAY	Proteoglycans in cancer	2.7 × 10^−2^	0.35
KEGG_PATHWAY	Dilated cardiomyopathy	3.4 × 10^−2^	0.38
KEGG_PATHWAY	Regulation of actin cytoskeleton	3.7 × 10^−2^	0.39
KEGG_PATHWAY	Calcium signaling pathway *	3.8 × 10^−2^	0.37

* Some of the known ASD-related pathways.

**Table 2:** The KEGG pathways enriched by the unique target genes in the ASD samples of NPC and neuron cells.

NPC ASD	Term	*p*-Value	Benjamini
KEGG_PATHWAY	Phosphatidylinositol signaling system *	4.3 × 10^−3^	0.6
KEGG_PATHWAY	ECM-receptor interaction *	8 × 10^−3^	0.57
KEGG_PATHWAY	Morphine addiction	0.01	0.51
KEGG_PATHWAY	Inositol phosphate metabolism	1.1 × 10^−2^	0.44
KEGG_PATHWAY	PI3K-Akt signaling pathway *	0.02	0.58
KEGG_PATHWAY	Circadian entrainment *	0.04	0.76
KEGG_PATHWAY	Focal adhesion *	4.7 × 10^−2^	0.76
KEGG_PATHWAY	Calcium signaling pathway *	4.8 × 10^−2^	0.72
**Neuron ASD**	**Term**	***p*** **-Value**	**Benjamini**
KEGG_PATHWAY	DNA replication	1.5 × 10^−3^	0.31
KEGG_PATHWAY	RNA transport	1.7 × 10^−3^	0.19
KEGG_PATHWAY	Mismatch repair	4.4 × 10^−3^	0.3
KEGG_PATHWAY	Protein processing in endoplasmic reticulum *	8.1 × 10^−3^	0.39
KEGG_PATHWAY	RNA degradation	1.2 × 10^−2^	0.44
KEGG_PATHWAY	Nucleotide excision repair	2.6 × 10^−2^	0.65
KEGG_PATHWAY	Spliceosome	2.8 × 10^−2^	0.63
KEGG_PATHWAY	Biosynthesis of antibiotics	5.4 × 10^−2^	0.81

* Some of the known ASD-related pathways.

## Data Availability

Data used in this study are available in the article.

## Supplementary Materials

The following are available at https://www.mdpi.com/article/10.3390/genes12121901/s1, Figure S1:(A) The percentages of differentially expressed genes in the reported ASD-associated KEGG pathways of the three cell types. (B) The expression levels of the gene members of the long-term depression pathway in the neuron cells of the normal(blue) and ASD (gold) cases. Figure S2: The size of regulatory cascades for normal and ASD in NPC and neural cells. Figure S3: The hierarchy clusters of the sizes of regulatory cascades for normal and ASD in the NPC stage. The color refers to the log2( size of a regulatory cascade), and the dark red represents the regulatory cascade lost in ASD. Figure S4: The hierarchy clusters of the sizes of regulatory cascades for normal and ASD in the neural cells. The color refers to the log2(size of a regulatory cascade), and the dark red represents the regulatory cascade lost in ASD. Figure S5: (A) GATA1 binds to the promoter regions of the two isoforms of the lncRNA ELF3-AS1. The promoter region of the lncRNA was defined as the window from 1000 bps upstream to 200 bps downstream from the transcription start site. (B) The spatial structures of the ELF3 and ELF3-AS1 in the human genome. Table S1. List of the differentially expressed lncRNAs in ASD samples of the three cell types. Table S2. The numbers of reference regulations in fetal brain that were confirmed by gene expression correlation. Table S3.The TFs that lost the interactions with their target genes in the ASD samples.
