# Supplementary material for: Gene Regulation Analysis Reveals Perturbations of Autism Spectrum Disorder during Neural System Development
Source: Genes (Basel). 2021 Nov 27;12(12):1901. doi: 10.3390/genes12121901 (PMC8700980; doi:10.3390/genes12121901)
Supplement: Supplementary file 1 [file genes-12-01901-s001.zip › genes-1442829-supplementary.pdf]

Supplementary Material

Supplementary Figures and Tables

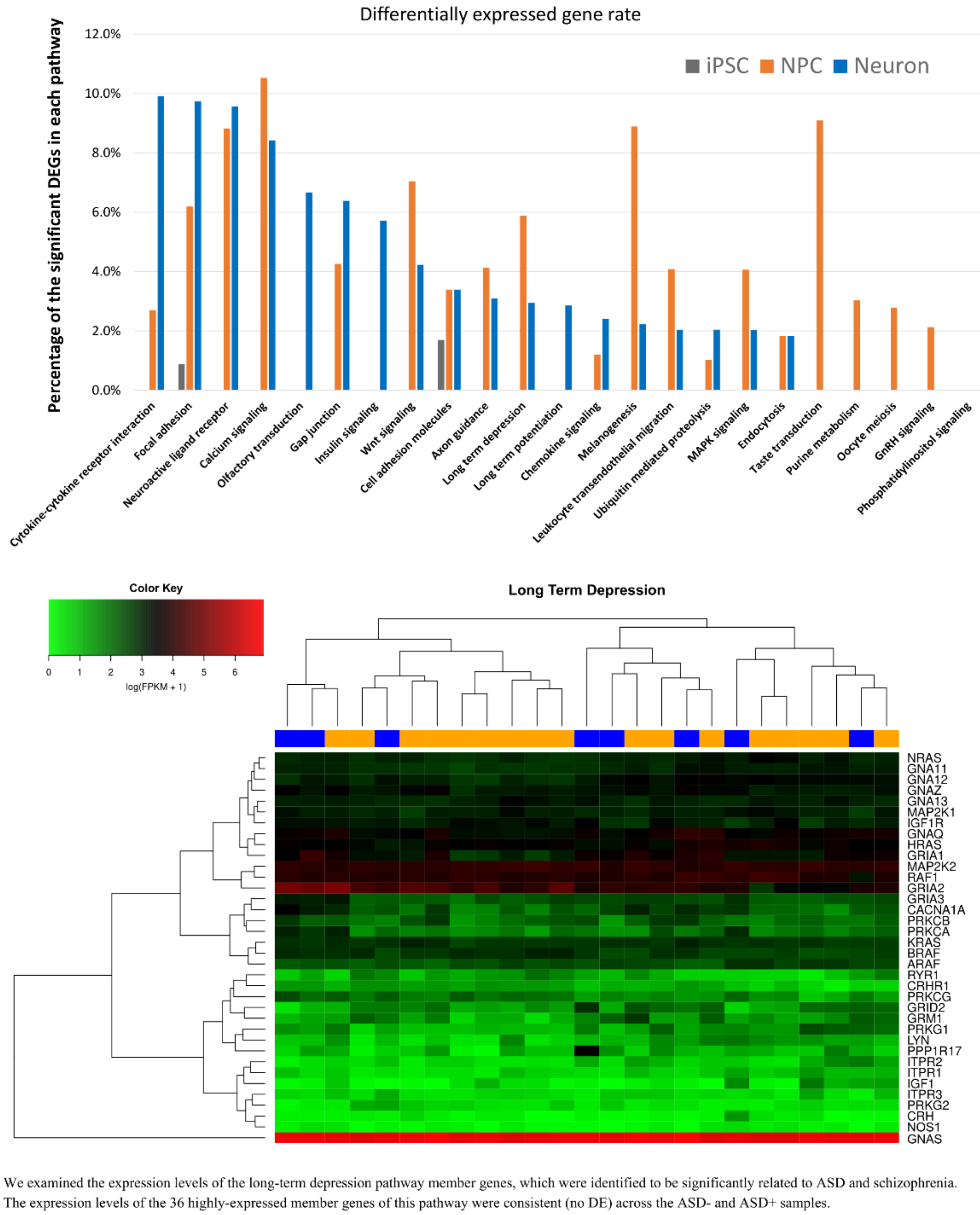

Supplementary Figure S1. (A) The differentially expressed gene rates of the reported ASD-associated KEGG pathways of the three cell types. (B) The expression levels of the gene members of the long-term depression pathway in the neuron cells of the normal(blue) and ASD (gold) cases.

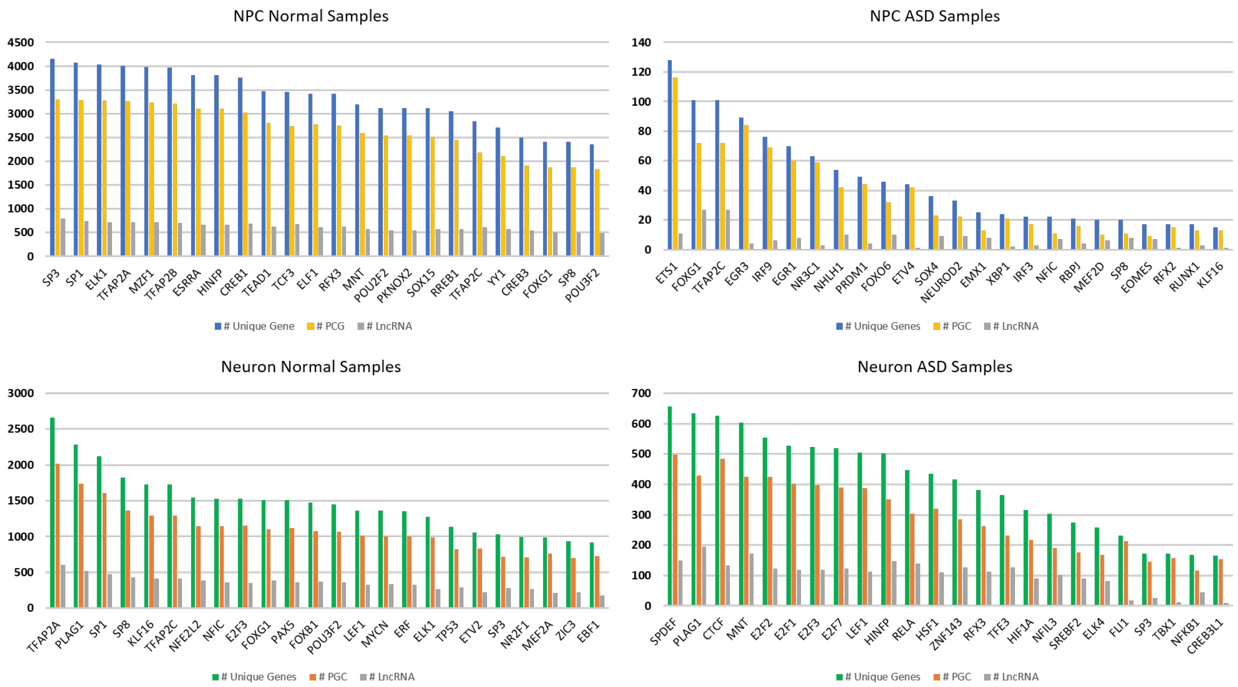

Supplementary Figure S2. The size of regulatory cascades for normal and ASD in NPC and neural cells.

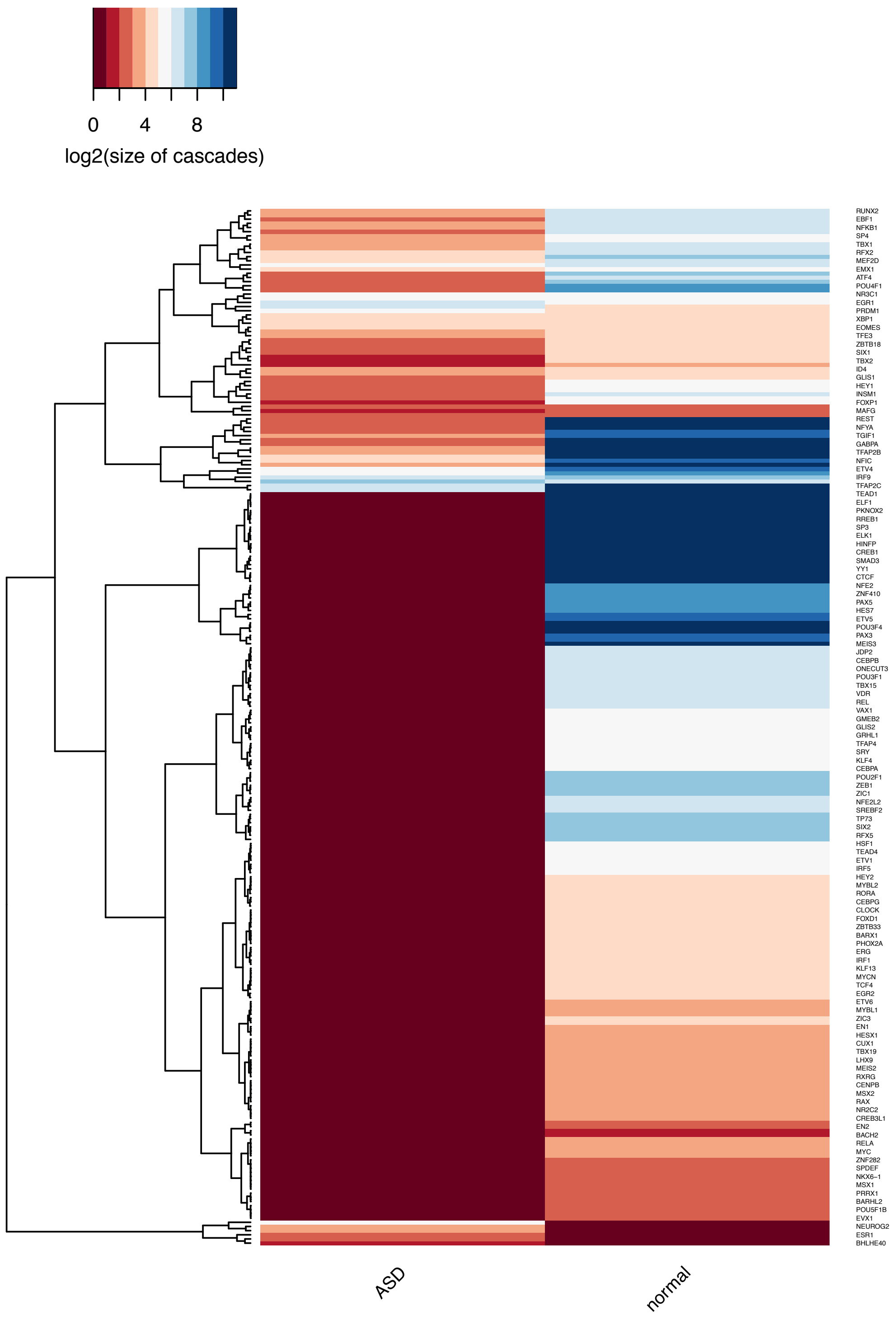

Supplementary Figure S3. The hierarchy clusters of the sizes of regulatory cascades for normal and ASD in the NPC stage. The color refers to the log2(size of a regulatory cascade), and the dark red represents the regulatory cascade lost in ASD.

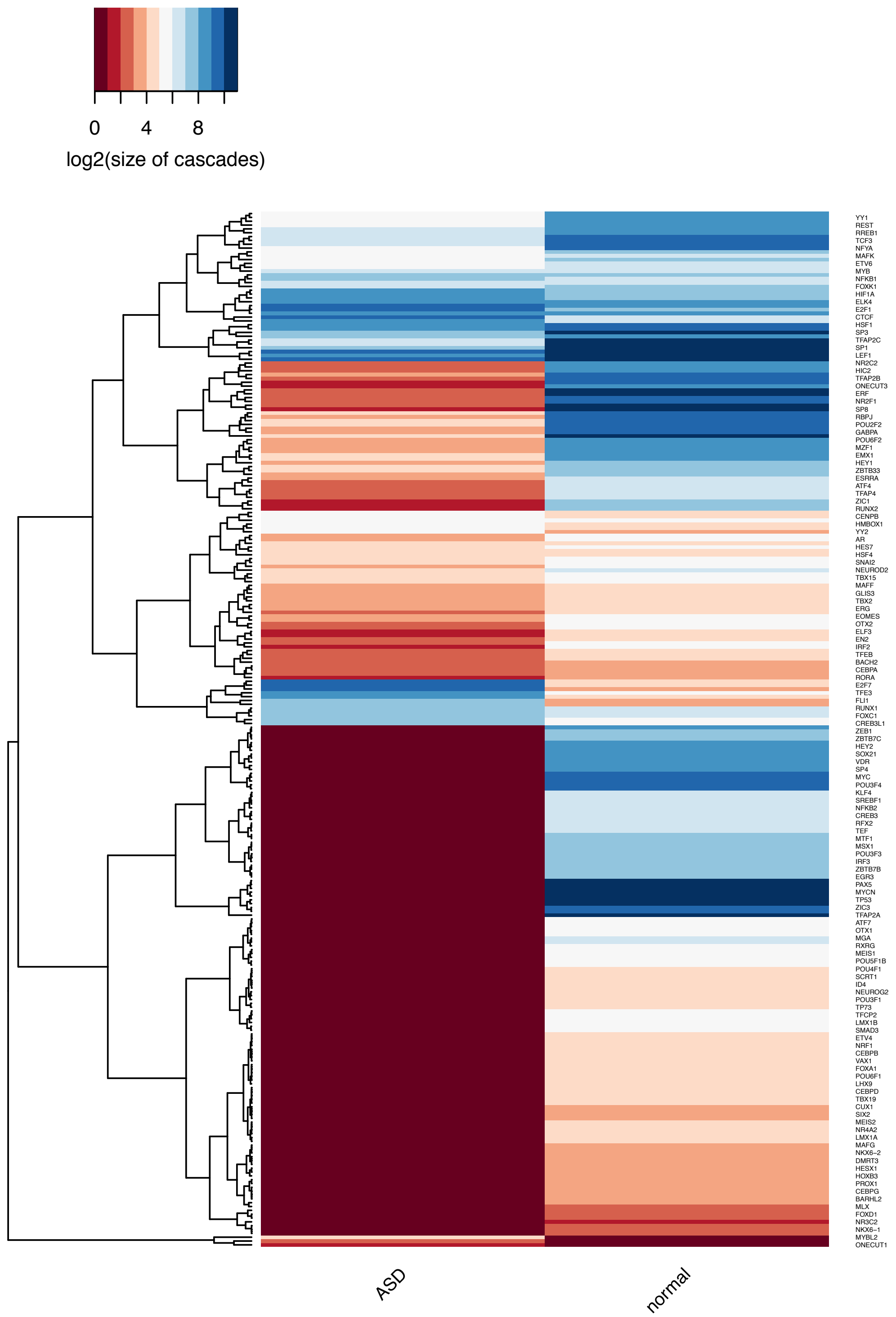

Supplementary Figure S4. The hierarchy clusters of the sizes of regulatory cascades for normal and ASD in the neural cells. The color refers to the log2(size of a regulatory cascade), and the dark red represents the regulatory cascade lost in ASD.

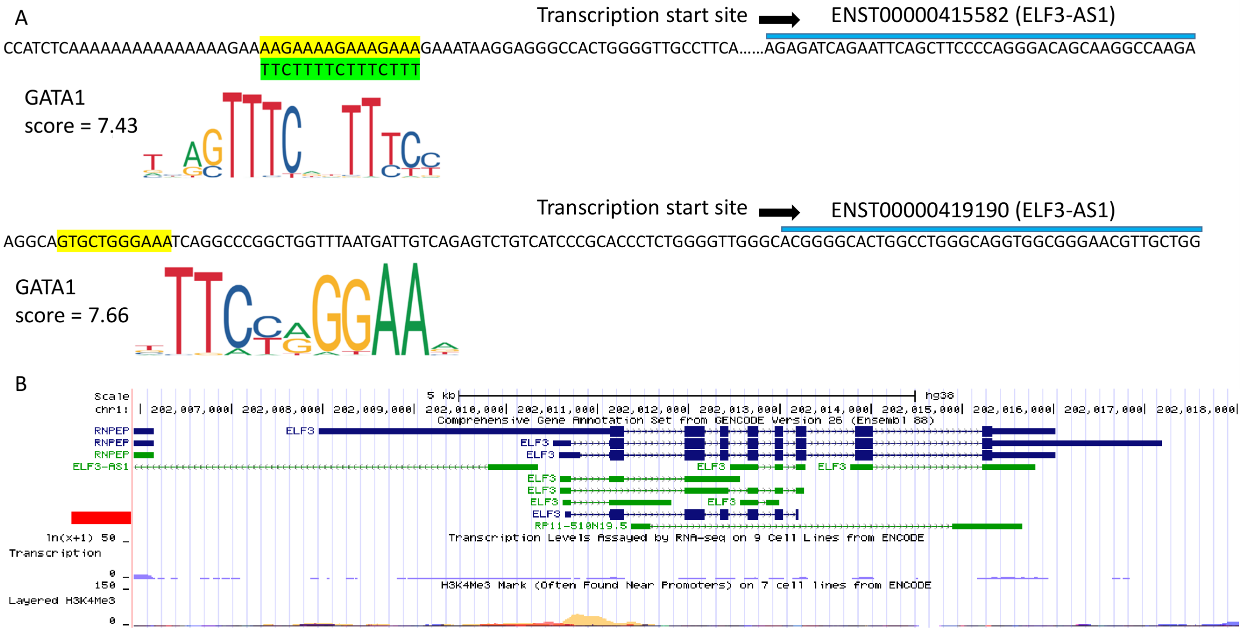

Supplementary Figure S5. (A) GATA1 binds to the promoter regions of the two isoforms of the lncRNA ELF3-AS1. The promoter region of the lncRNA was defined as the window from 1000 bps upstream to 200 bps downstream from the transcription start site. (B) The spatial structures of the ELF3 and ELF3-AS1 in the human genome.

Table S1. List of the differentially expressed lncRNAs in ASD samples of the three cell types.

**iPSC**	**Name**	**Type**	**logFC**	***p*-Value**	**FDR**
ENSG00000248587	GDNF-AS1	antisense_RNA	1.593235	2.65E-08	4.98E-05
ENSG00000234665	AL512625.3	lincRNA	-1.18237	1.98E-05	0.017664
ENSG00000231107	LINC01508	lincRNA	1.604464	2.44E-05	0.01877
ENSG00000230148	HOXB-AS1	antisense_RNA	1.609512	8.45E-05	0.049377
**NPC**	**Name**	**Type**	**logFC**	***p*-Value**	**FDR**
ENSG00000244342	LINC00698	lincRNA	3.120872	7.70E-23	1.00E-19
ENSG00000234165	AC114877.1	lincRNA	2.367867	6.58E-16	3.28E-13
ENSG00000198547	C20orf203	lincRNA	-1.73545	9.37E-13	3.11E-10
ENSG00000280780	JAKMIP2-AS1	antisense_RNA	1.988689	2.50E-12	7.43E-10
ENSG00000263571	AC004147.2	lincRNA	-1.70241	9.01E-12	2.50E-09
ENSG00000174403	C20orf166-AS1	antisense_RNA	-1.66264	1.99E-11	5.19E-09
ENSG00000122548	KIAA0087	lincRNA	-1.64027	5.80E-11	1.43E-08
ENSG00000269993	KC877982.1	lincRNA	1.815332	5.31E-10	1.06E-07
ENSG00000273771	AC024337.2	lincRNA	-1.49115	1.05E-09	1.98E-07
ENSG00000206532	AC117402.1	lincRNA	-1.44737	1.60E-09	2.88E-07
ENSG00000249464	LINC01091	lincRNA	1.562842	3.59E-08	4.95E-06
ENSG00000224969	AL645608.2	antisense_RNA	-1.35491	4.91E-08	6.56E-06
ENSG00000250616	AC012645.1	antisense_RNA	-1.26579	1.79E-07	1.97E-05
ENSG00000225472	AL136366.1	antisense_RNA	1.436728	3.39E-07	3.44E-05
ENSG00000253982	AC100810.1	antisense_RNA	-1.22235	1.28E-06	0.000108
ENSG00000260719	AC009133.3	antisense_RNA	1.405385	2.27E-06	0.00018
ENSG00000229847	EMX2OS	antisense_RNA	1.254132	3.01E-06	0.000232
ENSG00000249937	LINC02223	lincRNA	-1.1622	4.08E-06	0.000302
ENSG00000250742	LINC02381	lincRNA	-1.09098	5.24E-06	0.00038
ENSG00000250986	AC141928.1	lincRNA	1.29022	5.25E-06	0.00038
ENSG00000235597	LINC01102	lincRNA	-1.09494	5.65E-06	0.000406
ENSG00000231638	LUARIS	antisense_RNA	1.345416	6.16E-06	0.000435
ENSG00000245904	AC025164.2	antisense_RNA	-1.09411	6.42E-06	0.00045
ENSG00000231721	LINC-PINT	antisense_RNA	-1.10963	8.98E-06	0.000604
ENSG00000261136	AC023908.3	antisense_RNA	-1.04124	1.42E-05	0.000913
ENSG00000274021	AC024909.3	lincRNA	1.188693	1.53E-05	0.000965
ENSG00000236404	VLDLR-AS1	antisense_RNA	-1.04328	1.56E-05	0.000977
ENSG00000281881	SPRY4-IT1	lincRNA	1.189477	1.62E-05	0.001
ENSG00000274184	AC011815.2	lincRNA	-1.0815	1.64E-05	0.001006
ENSG00000230630	DNM3OS	antisense_RNA	-1.00848	2.53E-05	0.001486
ENSG00000270332	SMC2-AS1	lincRNA	-1.05136	5.48E-05	0.002832
ENSG00000229153	EPHA1-AS1	antisense_RNA	-1.19159	5.56E-05	0.002865
ENSG00000263958	AC091138.1	lincRNA	-1.02452	5.66E-05	0.002906
ENSG00000239911	PRKAG2-AS1	antisense_RNA	-1.1159	6.05E-05	0.00309
ENSG00000245067	IGFBP7-AS1	antisense_RNA	1.122918	6.59E-05	0.003316

ENSG00000215808	LINC01139	lincRNA	-1.01643	7.20E-05	0.003556
ENSG00000280707	AL353747.4	lincRNA	1.056588	0.000101	0.004678
ENSG00000224729	PCOLCE-AS1	antisense_RNA	-1.0042	0.000107	0.004862
ENSG00000224635	AL391095.1	lincRNA	1.039621	0.000134	0.005849
ENSG00000197182	MIRLET7BHG	lincRNA	1.119983	0.000228	0.008923
ENSG00000234614	AL450992.2	antisense_RNA	1.191283	0.000621	0.019451
ENSG00000284708	AL031731.1	antisense_RNA	-1.0411	0.000678	0.021049
**Neuron**	**Name**	**Type**	**logFC**	***p*-Value**	**FDR**
ENSG00000230630	DNM3OS	antisense_RNA	3.250921	9.63E-22	1.36E-18
ENSG00000278041	AL133325.3	lincRNA	-2.70107	5.33E-21	5.65E-18
ENSG00000280623	PCAT14	lincRNA	-2.04474	1.31E-13	4.02E-11
ENSG00000215808	LINC01139	lincRNA	-1.82521	4.57E-11	9.23E-09
ENSG00000213468	FIRRE	lincRNA	-1.77078	7.51E-11	1.46E-08
ENSG00000249669	CARMN	lincRNA	1.889543	2.27E-09	3.44E-07
ENSG00000223749	MIR503HG	lincRNA	1.799372	9.47E-09	1.33E-06
ENSG00000259485	LINC02253	lincRNA	1.855742	6.59E-08	7.45E-06
ENSG00000177406	AC021054.1	antisense_RNA	-1.64861	1.27E-07	1.35E-05
ENSG00000259370	AC103740.1	antisense_RNA	1.627037	1.77E-07	1.81E-05
ENSG00000266903	AC243964.2	antisense_RNA	-1.62902	1.96E-07	1.95E-05
ENSG00000267313	KC6	lincRNA	1.523084	6.09E-07	5.43E-05
ENSG00000266709	AC005224.4	lincRNA	1.487361	1.07E-06	8.96E-05
ENSG00000221819	GAS8-AS1	antisense_RNA	-1.35386	1.74E-06	0.000136
ENSG00000245067	IGFBP7-AS1	antisense_RNA	-1.24355	5.16E-06	0.000347
ENSG00000277954	AC092376.2	antisense_RNA	-1.19296	9.85E-06	0.000616
ENSG00000267506	AC021683.2	lincRNA	-1.29337	1.26E-05	0.000766
ENSG00000267530	LINC01836	lincRNA	-1.47	2.36E-05	0.001339
ENSG00000184809	B3GALT5-AS1	antisense_RNA	-1.13926	2.96E-05	0.001634
ENSG00000267466	AC021683.1	lincRNA	-1.23669	3.57E-05	0.001928
ENSG00000258545	RHOXF1-AS1	antisense_RNA	-1.12928	4.85E-05	0.002493
ENSG00000186960	LINC01551	lincRNA	-1.0809	6.11E-05	0.003055
ENSG00000250358	LINC02200	lincRNA	-1.10768	6.44E-05	0.003186
ENSG00000277013	AC008556.1	lincRNA	-1.27929	9.64E-05	0.004454
ENSG00000247157	LINC01252	lincRNA	-1.07639	9.67E-05	0.004454
ENSG00000261488	AC128688.2	antisense_RNA	-1.15373	0.000101	0.004639
ENSG00000177133	LINC00982	antisense_RNA	-1.04794	0.000104	0.00472
ENSG00000226476	LINC01748	lincRNA	-1.04763	0.00012	0.005359
ENSG00000232229	LINC00865	lincRNA	-1.21609	0.000147	0.006395
ENSG00000226702	MIR217HG	lincRNA	1.182371	0.000151	0.006556
ENSG00000231977	AL096828.1	lincRNA	1.232663	0.000233	0.00938

Table S2. The numbers of reference regulations in fetal brain that were confirmed by gene expression correlation.

**Correlation cutoff = 0.75 (PCGs)**	Normal sample	ASD sample	Overlap
iPSC	8,733	22,842	1,136
NPC	19,426	2,336	538
neuron	17,619	7,928	1,465
**Correlation cutoff = 0.75 (lncRNAs)**	Normal sample	ASD sample	Overlap
iPSC	3,944	3,967	200
NPC	8,110	573	110
neuron	8,031	2756	457

Table S3. The TFs that lost the interactions with their target genes in the ASD samples.

TF lost in ASD (NPC)	Number of genes	Number of protein-coding genes	Pathways	FDR
CREB1	3756	3023	Parkinson disease	3.33E-03
			Huntington disease	2.85E-03
CREB3	2494	1909	Parkinson disease	1.37E-05
			Ras Pathway	9.25E-04
			Huntington disease	7.51E-05
CTCF	2120	1660	Ubiquitin proteasome pathway	2.73E-02
			Parkinson disease	2.35E-02
			Huntington disease	3.30E-03
			EGF receptor signaling pathway	4.76E-02
ELF1	3419	2776	Parkinson disease	5.73E-03
			Huntington disease	8.37E-04
ELK1	4032	3268	Parkinson disease	5.47E-03
			Cytoskeletal regulation by Rho GTPase	4.94E-02
			Huntington disease	5.41E-03
ESRRA	3817	3106	Parkinson disease	1.15E-02
			Cytoskeletal regulation by Rho GTPase	3.95E-02
			Huntington disease	8.71E-03
HIF1A	2146	1685	Ubiquitin proteasome pathway	3.15E-02
			Parkinson disease	2.66E-02
			Huntington disease	3.78E-03
HINFP	3817	3106	Cytoskeletal regulation by Rho GTPase	3.95E-02
			Parkinson disease	1.15E-02
			Huntington disease	8.71E-03
MEIS3	1050	737	N/A	

MNT	3188	2588	Parkinson disease	4.58E-02
			Huntington disease	5.33E-03
MZF1	3988	3230	Cytoskeletal regulation by Rho GTPase	4.66E-02
			Parkinson disease	9.29E-03
			Huntington disease	7.17E-03
NRF1	1424	1126	Ubiquitin proteasome pathway	4.99E-03
			p53 pathway	1.84E-02
			Huntington disease	2.56E-03
PKNOX2	3116	2538	Parkinson disease	1.44E-02
			Huntington disease	1.83E-02
POU3F2	2359	1835	EGF receptor signaling pathway	4.08E-02
			Huntington disease	4.44E-02
			Gonadotropin-releasing hormone receptor pathway	6.17E-02
POU3F4	1357	995	Huntington disease	7.19E-03
RFX3	3412	2750	Parkinson disease	1.42E-02
			Huntington disease	1.98E-02
RREB1	3056	2446	Parkinson disease	1.16E-02
			Huntington disease	1.21E-03
SMAD3	2298	1877	Huntington disease	2.74E-02
SOX15	3110	2509	p53 pathway feedback loops 2	5.02E-02
			p53 pathway	4.25E-02
			Parkinson disease	1.29E-02
			Huntington disease	1.10E-02
SP1	4077	3282	Parkinson disease	5.54E-03
			Cytoskeletal regulation by Rho GTPase	2.07E-02
			Huntington disease	5.58E-03
SP3	4151	3302	Parkinson disease	5.79E-03

			Cytoskeletal regulation by Rho GTPase	3.23E-02
			Huntington disease	8.41E-03
			Parkinson disease	1.33E-02
TCF3	3457	2735	Huntington disease	2.02E-02
			Parkinson disease	1.62E-02
TEAD1	3469	2803	Huntington disease	9.43E-04
			Ubiquitin proteasome pathway	1.75E-03
			p53 pathway	1.30E-02
TFCP2	1387	1089	Huntington disease	3.08E-03
			Parkinson disease	5.34E-03
			Ras Pathway	3.83E-02
YY1	2713	2110	Huntington disease	3.09E-03
			Heme biosynthesis	4.56E-02
			Ubiquitin proteasome pathway	2.21E-02
			Transcription regulation by bZIP transcription factor	2.65E-02
			Parkinson disease	4.11E-02
			p53 pathway	4.71E-02
YY2	2065	1625	Huntington disease	9.84E-03
